# Optical Transmission Properties of Si_3_N_4_ Add-Drop Micro-Ring Resonator Induced by a Fabry–Perot Resonance Effect

**DOI:** 10.3390/s21196370

**Published:** 2021-09-24

**Authors:** Xinyang Chen, Linpeng Gu, Peijian Huang, Xuetao Gan, Ning Wang, Yong Zhu, Jie Zhang

**Affiliations:** 1 The Key Laboratory of Optoelectronic Technology & System, Education Ministry of China, Chongqing University, Chongqing 400044, China; 201908131063@cqu.edu.cn (X.C.); 20113269@cqu.edu.cn (P.H.); yongzhu@cqu.edu.cn (Y.Z.); zhangjie@cqu.edu.cn (J.Z.); 2 Key Laboratory of Light-Field Manipulation and Information Acquisition, Ministry of Industry and Information Technology, Xi’an 710129, China; gulinpeng@mail.nwpu.edu.cn (L.G.); xuetaogan@nwpu.edu.cn (X.G.); 3 Shaanxi Key Laboratory of Optical Information Technology, School of Science, Northwestern Polytechnical University, Xi’an 710129, China

**Keywords:** F-P cavity, optical transmission model, miscellaneous peaks, anti-reflection film

## Abstract

To resolve the problem of miscellaneous peaks and improve the accuracy of data processing in micro-ring resonators (MRRs), we propose an optical transmission model based on a Fabry–Perot (F-P) resonance effect in a Si_3_N_4_ add-drop MRR-waveguide structure, which is analyzed using a coupled mode theory and stationary interference method. The analysis indicates the experimentally obtained miscellaneous peaks are mainly induced by the multiple reflections between the two end grating couplers, which form a F-P cavity. In addition, an anti-reflection film on the interface of the grating couplers is proposed to reduce the F-P resonance effect. This work could be useful to analyze optical transmission properties of other MRR-based structures.

## 1. Introduction

Silicon photonics has become one of the most promising photonic integration platforms in recent years [1,2]. This can be mainly attributed to the combination of a very high index contrast and the availability of complementary metal-oxide-semiconductor (CMOS) fabrication technology, which allows the use of electronics fabrication facilities to make photonic circuitry [3,4,5]. As a typical integrated on-chip optical device, the micro-ring resonator (MRR) is widely used as a wavelength selective device in the field of sensing, e.g., temperature [6,7,8], humidity [9,10,11], and refractive index [12,13,14], due to its high-quality factor, narrow bandwidth, high integration, and other advantages. There are high requirements for the accuracy of the selected wavelength, including the peak intensity and position of the resonant peaks of MRR transmission. At this stage, there are a lot of works and experiments about the wavelength selection characteristics of MRR, and we notice that there are different degrees of miscellaneous peaks in the resonance spectrum of MRR transmission [15,16,17]. Compared with the resonance peaks of MRR, the miscellaneous peaks are disordered and relatively dense. The existence of these miscellaneous peaks inevitably affects the accuracy of wavelength selection, which leads to errors in the data analysis and processing results [18,19,20]. In the related experiments of MRR, Wirth et al. analyzed the source of the miscellaneous peaks and verified the existence of the Fabry–Perot (F-P) cavity [17]. Chao et al. analyzed the experimental results and showed that there is the F-P cavity effect, and compared with the transmission port, the background oscillation of the download port is smaller [18]. However, the corresponding work only states that the cause of the miscellaneous peaks may the formation of an F-P cavity between the waveguide grating couplers, and it lacks detailed theoretical analysis and experimental verification [17,18,19,20]. 

In this paper, we investigate the optical transmission properties of a Si_3_N_4_ add-drop MRR affected by a F-P cavity effect. The corresponding theoretical analysis and experimental measurements are carried out in detail. Meanwhile, we propose that an anti-reflection film on the interface (air and Si_3_N_4_) of the grating couplers can be useful to reduce the F-P cavity effect to the MRR output.

## 2. Theoretical Analysis

### 2.1. Analysis of an Add-Drop MRR without F-P Cavity Effect

**Figure 1:**
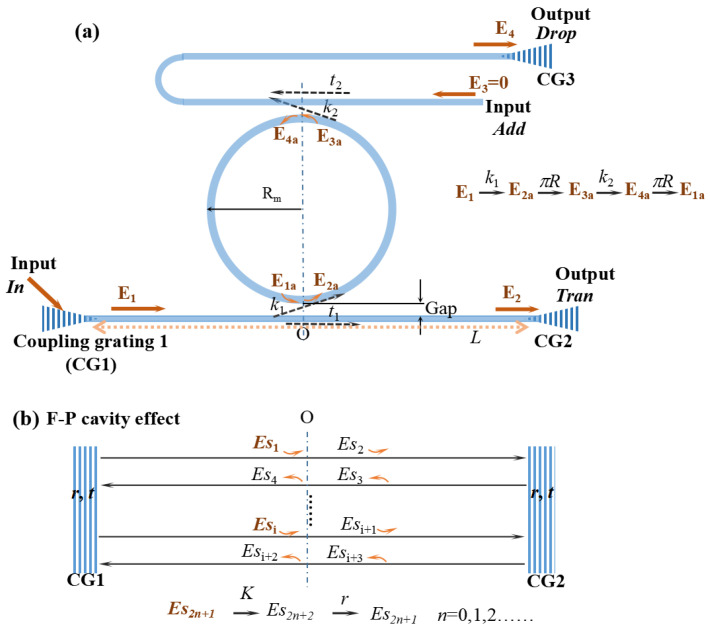
(**a**) An add-drop MRR schematic diagram. *CG1* and *CG2* are the coupling gratings. *In* is the optical signal input port; *Tran* is the direct output port; *Drop* is the download port; *Add* is the load port. The spacing between looped optical waveguide and straight optical waveguide is *Gap*, and the length of straight waveguide is *L*. (**b**) Optical transmission schematic diagram based on waveguide F-P cavity. *Es*_1_, *Es*_2_…*Es_n_* are optical amplitude at different stages. *r* and *t* are the reflection coefficient and transmission coefficient of grating coupler interface, respectively.

An add-drop MRR consists of a looped and a straight optical waveguide, as shown in Figure 1a. The optical signals are transmitted between the waveguides through evanescent wave coupling. The coupling resonance condition is expressed as:

(1) $$2n_{eff}\pi R_{m}=m\lambda_{m}$$

where *n_eff_* is the effective refractive index of the waveguide mode; *R_m_* is the micro-ring radius; *m* is the resonance series, taking a positive integer; *λ_m_* is the resonant wavelength.

*E*_1_, *E*_2_, *E*_3_, and *E*_4_ are the amplitude of signal *In*, *Tran*, *Add,* and *Drop*, respectively. *E*_1*a*_, *E*_2*a*_, *E*_3*a*_, and *E*_4*a*_ are the amplitude in MRR. *k*_1_, *k*_2_, *t*_1_, and *t*_2_ are the coupling coefficients and transmission coefficients of the two coupling regions, respectively, and they are assumed to satisfy *k*_1_^2^ + *t*_1_^2^ = 1 and *k*_2_^2^ + *t*_2_^2^ = 1, which means there are no losses in the coupling region. Based on the coupled mode theory, the transmission matrix is obtained:

(2) $$\left[\begin{array}{l} E_{1a}\\ E_{2a} \end{array}\right]=\left[\begin{array}{l} \frac{-t_{1}}{ik_{1}}\frac{1}{ik_{1}}\\ \frac{-1}{ik_{1}}\frac{t_{1}}{ik_{1}} \end{array}\right]\left[\begin{array}{l} E_{1}\\ E_{2} \end{array}\right]=M_{1}\left[\begin{array}{l} E_{1}\\ E_{2} \end{array}\right]$$

(3) $$\left[\begin{array}{l} E_{3a}\\ E_{4a} \end{array}\right]=\left[\begin{array}{l} 0\;\alpha^{1/2}p\\ {\left(\alpha^{1/2}p\right)}^{-1}\;0 \end{array}\right]\left[\begin{array}{l} E_{1a}\\ E_{2a} \end{array}\right]=M_{2}\left[\begin{array}{l} E_{1a}\\ E_{2a} \end{array}\right]$$

(4) $$\left[\begin{array}{l} E_{3}\\ E_{4} \end{array}\right]=\left[\begin{array}{l} \frac{-t_{2}}{ik_{2}}\frac{1}{ik_{2}}\\ \frac{-1}{ik_{2}}\frac{t_{2}}{ik_{2}} \end{array}\right]\left[\begin{array}{l} E_{3a}\\ E_{4a} \end{array}\right]=M_{3}\left[\begin{array}{l} E_{3a}\\ E_{4a} \end{array}\right]$$

(5) $$\left[\begin{array}{l} E_{3}\\ E_{4} \end{array}\right]=M_{3}M_{2}M_{1}\left[\begin{array}{l} E_{1}\\ E_{2} \end{array}\right]$$

where *α* = *exp* (*−γL_MRR_*) is the loss factor of one cycle, *L_MRR_* = 2*πR_m_* is the perimeter of the micro-ring, *γ* is the transmission loss coefficient of waveguide, *p* = *exp* (*iφ*) is the phase factor of half cycle, *φ* = 2*πn_eff_* (*πR_m_*)/*λ*. The relationship between optical amplitude *E_i_* and optical intensity *I_i_* can be expressed as:

(6) $$I_{i}=E_{i}\times E_{i}^{*}$$

where $E_{i}^{*}$ is the conjugate of *E_i_*. Then, the light intensity transmitted to the *Tran* and *Drop* can be expressed as:

(7) $$\frac{I_{2}}{I_{1}}=\frac{t_{2}^{2}\alpha^{2}-2t_{1}t_{2}\alpha cos\left(\phi \right)+t_{1}^{2}}{1-2t_{1}t_{2}\alpha cos\left(\phi \right)+{\left(t_{1}t_{2}\alpha \right)}^{2}}$$

(8) $$\frac{I_{4}}{I_{1}}=\frac{\left(1-t_{1}^{2}\right)\left(1-t_{2}^{2}\right)\alpha }{1-2t_{1}t_{2}\alpha cos\left(\phi \right)+{\left(t_{1}t_{2}\alpha \right)}^{2}}$$

where *ϕ* = 2*φ* is the phase of a cycle.

### 2.2. Analysis of an Add-Drop MRR with a F-P Cavity Effect

In real condition, the reflection coefficient of Si_3_N_4_ is not zero, there is inevitable multi-reflection phenomenon between two grating couplers. For the convenience of calculation, we only consider the reflection of the first interface of the grating coupler, that is, the signal only produces one reflection in the grating coupler. The optical transmission during the coupling region could be different from that mentioned in Section 2.1. Herein, we thought a F-P cavity is formed between two grating couplers. For simplicity, there are two suppositions: (1) the coupling occurs at point *O*; (2) the amplitude before point *O* and after point *O* is affected by parameter *K*, where *K* is the ratio of *E*_2_/*E*_1_ in the model without F-P cavity effect. According to the Equation (5), the ratio *K* is expressed as:

(9) $$K=\frac{t_{1}-t_{2}\alpha p^{2}}{1-t_{1}t_{2}\alpha p^{2}}$$

The transmission loss of the slab waveguide made of Si_3_N_4_ is 0.045 dB/m from ultraviolet (UV) to infrared radiation (IR) [21,22], which is almost transparent, so the transmission loss of the straight waveguide can be ignored. As shown in Figure 1b, there are two steps to calculate the real transmission properties. Firstly, the combined field at point *O* generated by multiple reflections (induced by the waveguide F-P cavity) is the actual incident field *E*_1_ of MRR. Secondly, we get the *Drop* signal according to the MRR transfer matrix mentioned in Section 2.1. *Es*_1_*, Es*_2_ … *Es_i_* are the transmission amplitudes at different reflections, respectively. The amplitude at point *O* coupled to the MRR can be expressed as:

(10) $$E_{O_{1}}=Es_{1}=Atexp\left(i\left(\delta_{0}-wt\right)\right)$$

(11) $$E_{O_{3}}=Es_{5}=AtKrKrexp\left(i\left(\delta_{0}+2\delta_{1}-wt\right)\right)=At{\left(Kr\right)}^{2}exp\left(i\left(\delta_{0}+2\delta_{1}-wt\right)\right)$$

(12) $$E_{O_{2}}=Es_{3}=AtKrexp\left(i\left(\delta_{0}+\delta_{1}-wt\right)\right)$$

(13) $$E_{O_{n}}=At{\left(Kr\right)}^{\left(n-1\right)}exp\left(i\left(\delta_{0}+\left(n-1\right)\delta_{1}-wt\right)\right)$$

where *A* is the amplitude of the incident light, *δ*_0_ is the initial phase, and *δ* is the phase difference of the transmission field at two consecutive *O* points, where *δ*_1_ = 2 *π*(*nL*)/*λ*. The sum of the combined field of *O*_1_, *O*_3_, *O*_5_ … *O_i_* (coupled to micro-ring) can be calculated by:

(14) $$E_{total}=E_{o_{1}}+E_{o_{3}}+E_{o_{5}}+\ldots =\left(At+AtK^{2}r^{2}exp\left(i2\delta_{1}\right)+AtK^{4}r^{4}exp\left(i4\delta_{1}\right)+\ldots \right)=\frac{At}{1-K^{2}r^{2}exp\left(i2\delta_{1}\right)}$$

Therefore, the equivalent incident intensity is expressed as follows:

(15) $$\begin{array}{l} I_{total}=E_{total}\times {E_{total}}^{*}=\frac{At}{1-\left(K^{2}\right)r^{2}exp\left(i2\delta_{1}\right)}\times \frac{At}{1-{\left(K^{2}\right)}^{*}r^{2}exp\left(-i2\delta_{1}\right)}\\ \;=\frac{I_{0}T}{1-\left[\left({\left(K^{2}\right)}^{*}+\left(K^{2}\right)\right)r^{2}\times cos\left(2\delta_{1}\right)+\left(\left(K^{2}\right)-{\left(K^{2}\right)}^{*}\right)r^{2}\times isin\left(2\delta_{1}\right)\right]+{\left(K^{2}\right)}^{*}\times \left(K^{2}\right)r^{4}}=\frac{I_{0}T}{1-\left[\xi_{2}+\xi_{1}\right]+\xi_{3}} \end{array}$$

where, *ξ*_1_, *ξ*_2_ and *ξ*_3_ is expressed as follows:

(16) $$\begin{array}{l} \zeta_{1}=\left(\left(K^{2}\right)-{\left(K^{2}\right)}^{*}\right)r^{2}\times isin\left(2\delta_{1}\right)\\ \;=\frac{sin\left(\phi \right)\times \left(-4t_{1}{}^{3}t_{2}\alpha +4t_{1}{}^{4}t_{2}{}^{2}\alpha^{2}cos\left(\phi \right)+4t_{1}t_{2}\alpha -4t_{1}{}^{3}t_{2}{}^{3}\alpha^{3}-4t_{2}{}^{2}\alpha^{2}cos\left(\phi \right)+4t_{1}t_{2}{}^{3}\alpha^{3}\right)}{1+4t_{1}{}^{2}t_{2}{}^{2}\alpha^{2}+t_{1}{}^{4}t_{2}{}^{4}\alpha^{4}-\left(4t_{1}t_{2}\alpha cos\left(\phi \right)\right)+\left(2t_{1}{}^{2}t_{2}{}^{2}\alpha^{2}cos\left(2\phi \right)\right)-\left(4t_{1}{}^{3}t_{2}{}^{3}\alpha^{3}cos\left(\phi \right)\right)}\times r^{2}\times sin\left(2\delta_{1}\right) \end{array}$$

(17) $$\begin{array}{l} \zeta_{2}=\left({\left(K^{2}\right)}^{*}+\left(K^{2}\right)\right)r^{2}\times cos\left(2\delta_{1}\right)\\ \;=\frac{\begin{array}{l} 2\times \left(t_{1}{}^{2}+4t_{1}{}^{2}t_{2}{}^{2}\alpha^{2}+t_{1}{}^{2}t_{2}{}^{4}\alpha^{4}\right)-4t_{1}{}^{3}t_{2}\alpha cos\left(\phi \right)+2t_{1}{}^{4}t_{2}{}^{2}\alpha^{2}cos\left(2\phi \right)-4t_{1}t_{2}\alpha cos\left(\phi \right)\\ -4t_{1}{}^{3}t_{2}{}^{3}\alpha^{3}cos\left(\phi \right)+2t_{2}{}^{2}\alpha^{2}cos\left(2\phi \right)-4t_{1}t_{2}{}^{3}\alpha^{3}cos\left(\phi \right) \end{array}}{1+4t_{1}{}^{2}t_{2}{}^{2}\alpha^{2}+t_{1}{}^{4}t_{2}{}^{4}\alpha^{4}-\left(4t_{1}t_{2}\alpha cos\left(\phi \right)\right)+\left(2t_{1}{}^{2}t_{2}{}^{2}\alpha^{2}cos\left(2\phi \right)\right)-\left(4t_{1}{}^{3}t_{2}{}^{3}\alpha^{3}cos\left(\phi \right)\right)}r^{2}\times cos\left(2\delta_{1}\right) \end{array}$$

(18) $$\xi_{3}={\left(K^{2}\right)}^{*}\times \left(K^{2}\right)\times r^{4}=K\times K^{*}\times K\times K^{*}\times R^{2}={\left(\frac{I_{2}}{I_{1}}\right)}^{2}\times R^{2}={\left(\frac{t_{2}^{2}\alpha^{2}-2t_{1}t_{2}\alpha cos\left(\phi \right)+t_{1}^{2}}{1-2t_{1}t_{2}\alpha cos\left(\phi \right)+{\left(t_{1}t_{2}\alpha \right)}^{2}}\right)}^{2}\times R^{2}$$

where *R* = *r*^2^ is the reflectivity and *T* = *t*^2^ is the transmissivity. According to Equations (7) and (8), the intensities of MRR *Drop* and *Tran* are expressed as:

(19) $$I_{Drop}=I_{total}\times \frac{I_{4}}{I_{1}}\times T=\frac{I_{0}T^{2}\left(1-t_{1}^{2}\right)\left(1-t_{2}^{2}\right)}{\left(1-\left[\xi_{2}+\xi_{1}\right]+\xi_{3}\right)\left(1-2t_{1}t_{2}cos\left(\phi \right)+{\left(t_{1}t_{2}\right)}^{2}\right)}$$

(20) $$I_{Tran}=I_{total}\times \frac{I_{2}}{I_{1}}\times T=\frac{I_{0}T^{2}\left(t_{2}^{2}-2t_{1}t_{2}cos\left(\phi \right)+t_{1}^{2}\right)}{\left(1-\left[\xi_{2}+\xi_{1}\right]+\xi_{3}\right)\left(1-2t_{1}t_{2}cos\left(\phi \right)+{\left(t_{1}t_{2}\right)}^{2}\right)}$$

Under ideal conditions, *R* = *r*^2^ = 0 and *T* = *t*^2^ = 1, there is no F-P cavity effect. Equations (15), (19) and (20) can be considered as the traditional MRR model mentioned in Section 2.1.

**Figure 2:**
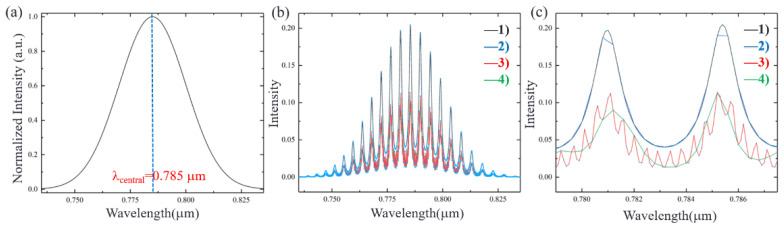
(**a**) Gaussian incident light with a center wavelength of 785 nm. (**b**) The output of Drop (1) without F-P cavity effect (Δ*λ* = 0.1 nm); (2) without F-P cavity effect (Δ*λ* = 0.4 nm); (3) with F-P cavity effect (Δ*λ* = 0.1 nm); (4) with F-P cavity effect (Δ*λ* = 0.4 nm). (**c**) Partial enlarged view in the wavelength range of 779 nm–787.5 nm.

To clearly show the analysis, simulations are carried out. According to the experimentally fabricated device, the parameters used in the simulation are as follows. The effective refractive index of Si_3_N_4_ is 2.2, MRR radius *R_m_* is 10 µm, *t*_1_ = *t*_2_ = 0.8, *L* = 300 µm, the transmissivity *T* is 0.7, the MRR loss factor *α* = 0.6, and the reflectivity *R* is 0.3 when the absorption loss of the interface is ignored. We set the wavelength resolution (Δ*λ*) in the simulation to be 0.1 nm and 0.4 nm (the wavelength resolution of the ocean spectrometer (USB-4000) used in our experiment in Section 3 is 0.4 nm). The incident light has a spectrum of a Gaussian line shape with a central wavelength of 785 nm, as shown in Figure 2a. Based on Equations (8) and (19), the output of *Drop* without and with F-P cavity effect (Δ*λ* = 0.1 nm and 0.4 nm) is shown in Figure 2b. It can be clearly seen from Figure 2c that when there is no F-P cavity effect, the wavelength selection is less affected by the wavelength resolution. When the F-P cavity effect exists, there are miscellaneous peaks in the *Drop* signal. By improving the wavelength resolution as Δ*λ* = 0.1 nm, the F-P cavity effect will be more obvious.

## 3. Experimental Results and Optimization

### 3.1. Experimental Results and Discussion

**Figure 3:**
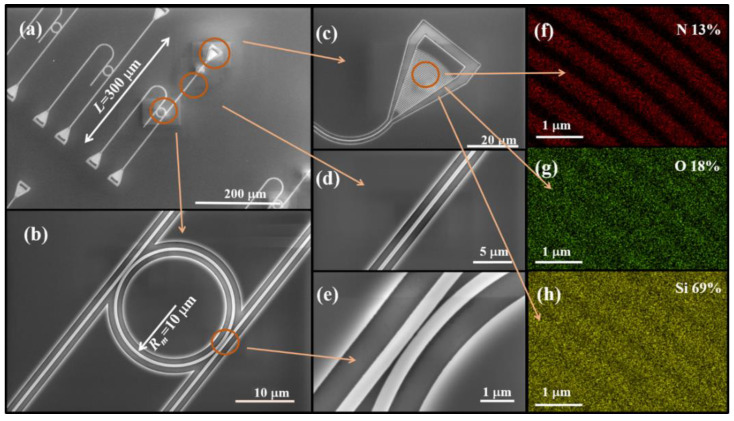
(**a**–**e**) The SEM images of the fabricated Si_3_N_4_ waveguide structure. (**f**–**h**) The EDS images of the fabricated Si_3_N_4_ waveguide structure.

To verify the above theoretical analysis, add-drop MRR samples with different radii and coupling gaps with the bus-waveguide were fabricated with electron beam lithography and reaction ion etching. Figure 3a–e present the scanning electron microscope (SEM) images of the fabricated Si_3_N_4_ waveguide structure. The length of the waveguide *L* is 300 µm ± 3 µm and the radius *R_m_* is 10 µm ± 0.1 µm. Figure 3f–h are the energy depressive spectroscopy (EDS) images of the fabricated Si_3_N_4_ waveguide structure material. The results show that the waveguide material is Si_3_N_4_ and the substrate material is SiO_2_. The mass percentages of N, O, and Si in the measured grating region are 13%, 18%, and 69%. 

**Figure 4:**
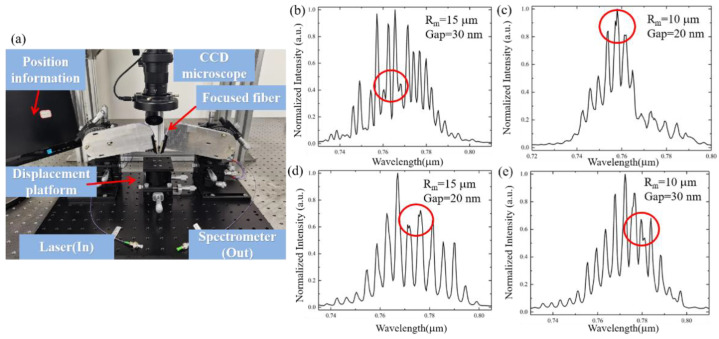
(**a**) Schematic diagram of test platform. (**b**) *R_m_* = 15 µm, *Gap* = 30 nm, (**c**) *R_m_* = 10 µm, *Gap* = 20 nm, (**d**) *R_m_* = 15 µm, *Gap* = 20 nm. (**e**) *R_m_* = 10 µm, *Gap* = 30 nm.

The experimental system is shown in Figure 4a. A broadband light source is coupled to the waveguide grating coupler using a focused fiber. The output is also coupled to the spectrometer orderly using a waveguide grating coupler and a focused fiber. The spectrometer is an ocean spectrometer with a wavelength resolution of 0.4 nm. A charge coupled device (CCD) microscope is used to determine the position information of focused fiber and waveguide.

We have measured 198 samples. Herein, four representative experimental results (*Drop*) are shown in Figure 4b–e. We can observe the miscellaneous peaks phenomenon through the experimental results, but the details cannot be clearly observed due to the insufficient resolution of the spectrometer used in the experiment. Therefore, based on Equation (15), we simulate the equivalent incident light of F-P cavity effect with higher wavelength resolutions for further analysis.

**Figure 5:**
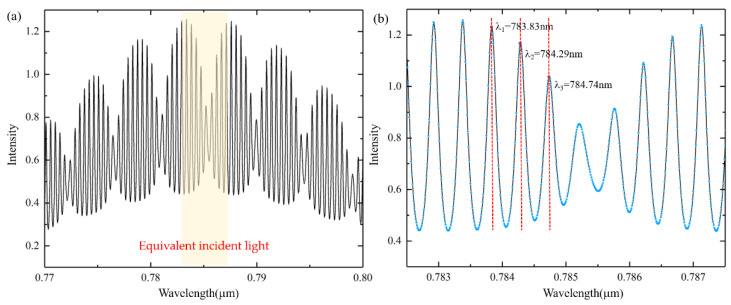
(**a**) The equivalent incident light *I_total_* of F-P cavity effect (wavelength resolution Δ*λ* = 0.01 nm). (**b**) Detailed information about the wavelength range of 782.5 nm–787.5 nm with a wavelength resolution of 0.01 nm.

The wavelength resolution is improved to 0.01 nm. The simulation results of the equivalent incident light of F-P cavity effect in the wavelength range of 770–800 nm are shown in Figure 5a. Figure 5b shows the detailed information of the wavelength range of 782.5–787.5 nm when Δ*λ* is 0.01 nm. The simulation results show that the equivalent incident light of F-P cavity effect has free spectral range (FSR) and is the same as the standard F-P cavity formed by the two end grating couplers. With the same parameters, the FSR of the equivalent incident light and standard F-P cavity is equal to 0.46 nm (*λ*_1_ = 783.83 nm). 

**Figure 6:**
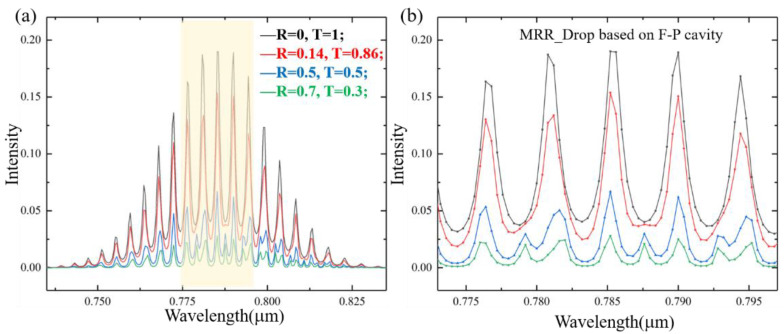
(**a**) Spectral signal of *Drop* under different reflectivities. *R* = 0 (black), *R* = 0.14 (red), *R* = 0.5 (blue) and *R* = 0.7 (green). (**b**) Partial enlarged view at resonance wavelength.

The F-P cavity effect will be different due to different interface reflectivities of grating couplers. To characterize the F-P cavity effect, we set *γ*(*λ*) to represent the intensity error value at the resonance wavelength, *γ*(*λ*) = (*I_MRR_* − *I_FP-MRR_*)/*I_MRR_*, where *I_FP-MRR_* and *I_MRR_* are the signal intensity with and without F-P cavity effect, respectively. The intensity error value of multiple resonance wavelengths is calculated by *γ*(*R*) = (*γ*(*λ*_1_) + *γ*(*λ*_2_) + … *γ* (*λ_n_*))/*n*. We simulate the *Drop* signal under different reflectivities *R* (0/0.14/0.5/0.7), where *R* = 0.14 is the reflectivity of the interface between air and Si_3_N_4_, and the results are shown in Figure 6.

Here, we select five resonance wavelengths in the signal center, which are *λ* = 776.57, 780.96, 785.40, 789.89, and 794.43 nm. From the simulation results, it can be seen that when *R* = 0.14, 0.5, and 0.7, *γ* (0.14) = 27.07%, *γ* (0.5) = 70.55%, and *γ* (0.7) = 87.61%. When *R* = 0.5 and *R* = 0.7, we can hardly distinguish the position of its resonance wavelength, and MRR cannot play a role in wavelength selection. When the reflectivity decreases, the influence of the waveguide F-P cavity on the MRR transmission signal decreases. According to the analysis, the F-P cavity has a great influence on the wavelength selection characteristics of the MRR. When the interface of the coupled grating has a large reflectivity, the position of the resonance peak cannot even be distinguished.

### 3.2. Optimization Scheme

**Figure 7:**
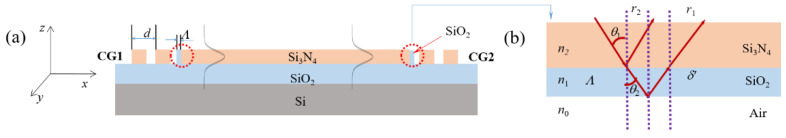
(**a**) Anti-reflection film on the side wall of the grating coupler. (**b**) Schematic diagram of interface reflection. *n*_0_ = 1; *n*_1_ = 1.45; *n*_2_ = 2.4.

A layer of anti-reflection film with a refractive index between air and Si_3_N_4_ is introduced on the side wall of the grating coupler (there is the same procession to CG2), as shown in Figure 7a. The grating period is *d*, and the thickness of the anti-reflection film is Λ. Figure 7b shows that the phase difference between the two reflected lights is *δ*′ (*δ*′ = 2*π* (2*n*Λ)/*λ*). The reflection coefficients at the two interfaces are *r*_1_ and *r*_2_, which are expressed as:

(21) $$r_{1}=\frac{n_{1}-n_{0}}{n_{1}+n_{0}},\;r_{2}=\frac{n_{2}-n_{1}}{n_{2}+n_{1}}$$

The intensity combination *I*′ of the reflected light *I*_1_′ and *I*_2_′ can be expressed as:

(22) $$I^{\prime }={I_{1}}^{\prime }+{I_{2}}^{\prime }+2\sqrt{{I_{1}}^{\prime }{I_{2}}^{\prime }}cos\left(\frac{4\pi }{\lambda }n\Lambda \right)$$

When the optical thickness of the anti-reflection film is one quarter of a certain wavelength, *δ*′ = *π*, then the directions of the two vectors are opposite, and the vector combination is the smallest. Under ideal conditions, *r*_1_ = *r*_2_, the combined reflection coefficient is the smallest. At this time *r*′ = 0 (*R*′ = 0), *n*_1_ can be expressed as:

(23) $$n_{1}=\sqrt{n_{0}n_{2}}$$

**Figure 8:**
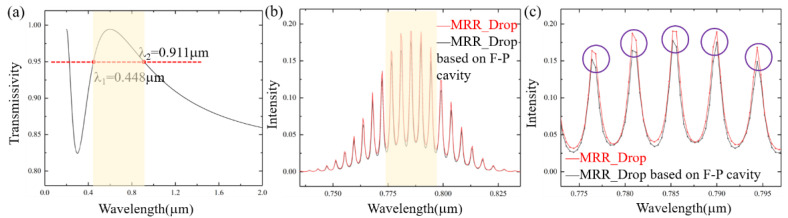
(**a**) Transmission spectrum after depositing 150 nm SiO_2_ anti-reflection film. (**b**) When transmittance is 0.95, comparison of *Drop* signal with and without F-P effect. (**c**) Partial enlarged view of resonance peak signal.

According to the air refractive index (*n*_0_ = 1) and Si_3_N_4_ refractive index (*n*_2_ = 2.4), the ideal refractive index of anti-reflection film is 1.549. However, in fact, we cannot find the material with exactly the same refractive index, so we use SiO_2_ as the material to prepare the film, and *n_SiO_*_2_ is 1.45. According to Equation (19), we set the thickness of SiO_2_ anti-reflection film as 150 nm, and the simulation results of its transmission spectrum are shown in Figure 8a. When the wavelength range is 0.448~0.911 µm, the *R*′ is less than 0.05. According to Equation (16), when *R*′ = 0.05, the simulation results of *Drop* signal with and without F-P cavity effect are shown in Figure 8b, and the partial enlarged view is shown in Figure 8c. According to Section 3.1, the resonance wavelength intensity error value *γ* (0.05) is 11.08%, and the influence of waveguide F-P on the MRR output signal is almost nonexistent.

## 4. Conclusions

An optical transmission model based on a waveguide Fabry–Perot (F-P) cavity effect in an add-drop MRR waveguide structure was proposed and analyzed in detail. The experimental results show the phenomenon of miscellaneous peaks induced by the multiple reflections between the two end grating couplers, and the FSR can be observed after the resolution is improved. In addition, the F-P cavity effect will be different due to the different interface reflectivities. Meanwhile, according to the principle of interference cancellation, we propose that an anti-reflection film on the interface of the grating couplers can be useful to reduce the F-P cavity effect to the MRR output. The simulation results show that when a SiO_2_ film with an optical thickness of 150 nm is used, a transmittance greater than 95% can be achieved in the wavelength range 0.448~0.911 µm. Combined with the optical transmission model based on a waveguide F-P cavity effect, the intensity error value at resonance wavelength *γ* = 11.08%, and the spectral signal of MRR *Drop* is almost not affected by the waveguide F-P cavity. This work could be useful to improve optical transmission properties of other waveguide structures.

## Data Availability

Not applicable.
